# Severe Clinical Worsening in COVID-19 and Potential Mechanisms of Immune-Enhanced Disease

**DOI:** 10.3389/fmed.2021.637642

**Published:** 2021-06-22

**Authors:** John P. Hussman

**Affiliations:** Hussman Foundation, Ellicott City, MD, United States

**Keywords:** COVID-19, clinical deterioration, monocytes/macrophages, antibodies, vaccines

## Abstract

Infection by the novel SARS-CoV-2 coronavirus produces a range of outcomes, with the majority of cases producing mild or asymptomatic effects, and a smaller subset progressing to critical or fatal COVID-19 disease featuring severe acute respiratory distress. Although the mechanisms driving severe disease progression remain unknown, it is possible that the abrupt clinical deterioration observed in patients with critical disease corresponds to a discrete underlying expansion of viral tropism, from infection of cells comprising respiratory linings and alveolar epithelia to direct infection and activation of inflammatory monocytes and macrophages. Dysregulated immune responses could then contribute to disease severity. This article discusses the potential role of monocyte/macrophage (Mo/Mϕ) infection by SARS-CoV-2 in mediating the immune response in severe COVID-19. Additional mechanisms of immune-enhanced disease, comprising maladaptive immune responses that may aggravate rather than alleviate severity, are also discussed. Severe acute clinical worsening in COVID-19 patients may be influenced by the emergence of antibodies that participate in hyperinflammatory monocyte response, release of neutrophil extracellular traps (NETs), thrombosis, platelet apoptosis, viral entry into Fc gamma receptor (FcγR)-expressing immune cells, and induction of autoantibodies with cross-reactivity against host proteins. While the potential roles of Mo/Mϕ infection and immune-enhanced pathology in COVID-19 are consistent with a broad range of clinical and laboratory findings, their prominence remains tentative pending further validation. In the interim, these proposed mechanisms present immediate avenues of inquiry that may help to evaluate the safety of candidate vaccines and antibody-based therapeutics, and to support consideration of pathway-informed, well-tolerated therapeutic candidates targeting the dysregulated immune response.

## Introduction

The SARS-CoV-2 coronavirus emerged in late-2019 in Wuhan, China, presenting as pneumonia of unknown etiology. The virus was isolated on January 7, 2020, and its genetic sequence was published 5 days later. Within 10 weeks, the associated disease, COVID-19, was declared a global pandemic by the World Health Organization (WHO). As of December 2020, 65 million confirmed or probable cases of SARS-CoV-2 infection have been identified, with over 1.5 million fatalities (1). Severe clinical outcomes and fatalities among a subset of symptomatic COVID-19 patients have created an urgent need for the development of safe and effective vaccines and therapeutics.

Infection by the novel SARS-CoV-2 coronavirus results in multi-modal outcomes, with the majority of cases producing mild or asymptomatic effects, and a smaller subset progressing to critical or fatal COVID-19 disease featuring severe acute respiratory distress. While the mechanisms driving severe disease progression remain unknown, it is possible that the abrupt clinical deterioration observed in patients with critical disease corresponds to a discrete underlying expansion of viral tropism, from infection of cells comprising respiratory linings and alveolar epithelia to direct infection and activation of inflammatory monocytes and macrophages. Direct viral infection of these cells can promote a transcriptional shift toward invasive and inflammatory phenotypes, consistent with those observed in severe COVID-19. This shift may coincide with the induction of antibodies that participate in immune-enhanced disease severity.

We begin by describing several immune hallmarks of mild vs. severe COVID-19, with an emphasis on the contribution of inflammatory monocyte/macrophage (Mo/Mϕ) subsets to features observed in patients with severe disease. Potential mechanisms of Mo/Mϕ infection and immune-enhanced disease progression are discussed. “Immune enhancement” in this context refers to maladaptive immune responses that may aggravate rather than alleviate disease severity, beyond cytopathic effects of the virus. Attention is given to the emergence of neutralizing IgG antibodies directed against the SARS-CoV-2 spike, and their potential contribution to hyperinflammatory monocyte response, release of neutrophil extracellular traps (NETs), thrombosis, platelet apoptosis, and antibody-dependent enhancement (ADE) of viral entry into Fc gamma receptor (FcγR)-expressing immune cells. Features of antibody response that may be relevant in the evaluation of vaccine safety are described, including IgG fucosylation, potential generation of autoantibodies with cross-reactivity to host proteins, and interactions of antigen–antibody immune-complexes with Fcγ receptors and components of the complement pathway. Signal transduction pathways, particularly downstream of viral pattern-recognition receptors (PRRs) and FcγRs, are also discussed in the context of immune-enhanced pathology and possible therapeutic modulation.

The potential roles of monocyte/macrophage infection and immune-enhanced pathology in COVID-19 are consistent with a broad range of evidence, but their prominence remains tentative pending further validation. In the interim, these proposed mechanisms present specific points of investigation that may be of immediate benefit in the testing of candidate biological interventions. The development of safe and effective vaccines and antibody-based therapeutics relies on evaluation to limit the possibility of immune-enhanced disease. Clinical treatment of active cases may also benefit from the consideration of pathway-informed, well-tolerated therapeutic candidates targeting mediators of the maladaptive immune response.

## Immune Correlates of Disease Progression in Covid-19

Immune defense against viral pathogens involves the coordination of immediate innate and later pathogen-specific adaptive responses that promote viral recognition, containment, clearance, and host immunological memory. Entry of enveloped viruses such as coronaviruses into host target cells is achieved by binding of a viral surface protein to a receptor protein on the host cell membrane, followed by membrane fusion or endocytosis, and introduction of the viral genome into the host cell. Viral components and genetic material are sensed by host innate PRRs such as Toll-like (TLR), NOD-like (NLR), C-type lectin (CLR), and RIG-I-like (RLR) receptors. Downstream signaling cascades promote the transcription of interleukin (IL)-1, IL-18, Type-I (α/β), -II (γ), and -III (λ) interferons (IFNs), a large set of IFN-stimulated genes (ISGs), inflammatory cytokines such as TNF-α, IL-12, and IL-6, and leukocyte chemoattractants such as CCL2/MCP-1 and CCL3/MIP-1a. These molecules act to impede viral replication, and to recruit cytolytic immune cells such as natural killer (NK) cells and neutrophils, as well as phagocytes such as monocyte-derived macrophages and dendritic cells (DC). Complement proteins contribute to the inactivation of viruses, and phagocytes ingest and present viral antigens, via major histocompatibility complex (MHC) molecules, to cells of the adaptive immune system. These interactions promote expanded populations of pathogen-specific CD4+ T-helper cells, CD8+ cytotoxic T-lymphocytes, antibody-producing B-cells, and T and B memory cells (2).

COVID-19 produces varied levels of disease severity in infected individuals. The majority of infections with SARS-CoV-2 produce mild or asymptomatic outcomes. Based on large-scale, unbiased testing, it is estimated that 40–45% of individuals infected with SARS-CoV-2 are asymptomatic (3). Close to half of these cases are reported to show lung abnormalities such as ground-glass opacities and consolidation based on CT imaging. Some individuals who are asymptomatic at the time of a positive test may become symptomatic later; these individuals can potentially be distinguished by elevated levels of lactate dehydrogenase (LDH) during the pre-symptomatic phase (4). While viral shedding by asymptomatic or pre-symptomatic individuals may account for close to half of SARS-CoV-2 transmission (5), the prevalence of mild outcomes among individuals in low-risk groups may confound containment efforts. Among symptomatic cases, approximately 81% are classified as having mild disease featuring mild or absent pneumonia, 14% having severe disease featuring respiratory distress, lung infiltrates, or low oxygen saturation, and 5% having critical disease including acute respiratory failure, septic shock, or multi-organ failure (6).

Progression to severe COVID-19 disease is associated with a variety of alterations in immune cell populations and inflammatory response. Asymptomatic presentation in COVID-19 is reported to be associated with a high prevalence of NK cells, with severely diseased patients not requiring ICU treatment having significantly higher NK cell counts than ICU patients (7). The onset of symptoms in COVID-19 is accompanied by a rapid increase in “classical” CD14+CD16– monocytes expressing the sialic acid-binding immunoglobulin-like lectin CD169/Siglec-1. These monocytes are observed with significantly higher frequency in patients with mild disease, relative to those with severe disease, and express IFN-γ and monocyte chemoattractant protein CCL8/MCP-2 (8). The functional roles of CD169 include sialic-acid based pattern recognition and maintenance of immunological tolerance. CD169 also helps to bridge the innate and adaptive immune response by facilitating the capture and presentation of viral particles to invariant NK T-cells, CD8+ T-cells and B-cells (9).

In COVID-19 patients stratified by disease course, longitudinal profiling of peripheral blood samples identifies the most significant changes to be in the function and proliferation of monocytes (10). Inflammatory monocytes and monocyte-derived macrophages are abundant in the lungs of patients with severe COVID-19 (11). Increased proliferation of “intermediate” CD14+CD16+ monocytes during severe disease progression is associated with a corresponding reduction in NK cell frequency (7). Elevated levels of inflammatory CD14+CD16+ monocytes expressing costimulatory protein CD80/B7, the hemoglobin-haptoglobin scavenger receptor CD163, and lysosomal proteins CD68/LAMP-2 and CD208/LAMP-3 are predictive of severe disease and ICU admission (12). Increased proliferation of CD14+CD16 monocytes is also coupled with a near-universal reduction of antigen-presentation molecules CD86 and HLA-DR in monocytes of patients with severe disease (13). These changes suggest that severe COVID-19 may be mediated by transcriptional changes in Mo/Mϕ subsets that favor pro-inflammatory and cytotoxic functions.

In patients with severe COVID-19, a marked shift in monocyte populations toward cells expressing the Fc-gamma III receptor CD16/FcγRIII is accompanied by expression of chemoattractants including macrophage inflammatory proteins CCL3/MIP-1a, CCL4/MIP-1b, and CCL23/MIP-3 (8). Increased monocyte expression of the proliferation marker Ki-67 is observed, and strongly correlates with levels of C-reactive protein (CRP) (10). Increased levels of the chemokine CXCL10/IP-10 are also observed in nearly all COVID-19 patients, but unlike moderately diseased patients, in which high IP-10 levels are transient, severely diseased patients maintain elevated levels that are proportional to disease progression (13).

Severe/critical COVID-19 pneumonia is associated with significant elevation of inflammatory cytokine release, including IL-6, IL-2R, IL-8, and TNF, as well as the anti-inflammatory cytokine IL-10, and chemoattractants that mediate leukocyte recruitment, particularly CXCL10/IP-10, CCL2/MCP-1, and CCL3/MIP-1a. Hallmarks of severe infection include elevated acute phase markers such as CRP, serum ferritin, LDH, D-dimer, and procalcitonin. Severe cases feature elevated neutrophil counts and depressed lymphocyte counts, resulting in a significantly higher neutrophil-to-lymphocyte ratio (NLR) relative to non-severe cases. Age is a significant risk factor, with a low incidence of symptomatic cases in children and young adults (< age 24). Chronic diseases such as diabetes, hypertension, cardiovascular disease, and chronic obstructive pulmonary disease confer additional risk, although only half of severe cases feature these predisposing factors (14, 15).

Taken together, increasing severity in COVID-19 appears to be associated with a reduction in NK cells, profound lymphopenia, increased proliferation and activation of CD14+CD16+ inflammatory monocytes with reduced antigen-presentation markers, increased cytokine release, elevated acute-phase reactants, and expression of chemokines that mediate the recruitment of inflammatory monocytes, macrophages, and neutrophils to infected tissue.

Notably, severe disease cases resulting from infection by the related SARS-CoV (Severe Acute Respiratory Syndrome) and MERS-CoV (Middle East Respiratory Syndrome) coronaviruses feature similar inflammatory infiltrates, elevated cytokine and chemokine release, and respiratory distress marked by diffuse alveolar damage (DAD) (16).

## Severe Clinical Worsening in Covid-19

A commonly reported feature of severe COVID-19 is an abrupt deterioration in clinical condition characterized by rapid progression to respiratory distress, with elevation of acute phase reactants and inflammatory mediators resembling those observed in cytokine release syndrome (CRS) and macrophage activation syndrome (MAS)/secondary hemophagocytic lymphohistiocytosis (HLH) (17). Several features of progression to severe disease, including hyperinflammation, cytokine release, and dysregulated coagulation suggest that macrophage activation may contribute to COVID-19 pathology (18, 19).

It is possible that the abrupt clinical deterioration observed in patients with critical disease corresponds to an expansion of viral tropism, from infection of cells comprising respiratory linings and alveolar epithelia to direct infection and activation of inflammatory monocytes and macrophages (Mo/Mϕ). In the present context, infection of Mo/Mϕ describes viral entry characterized by escape from lysosomal degradation, with a resulting capacity to amplify, inhibit, or otherwise reprogram cellular activities. These potential responses include IFN-independent cytokine/chemokine release triggered by endosomal or intracellular PRRs, transcription of the viral genome, and even support of productive viral replication.

Monocytes and macrophages have been demonstrated to mediate the persistence or spread of viruses belonging to 13 different families, including coronaviruses. Mo/Mϕ have high phagocytic activity, and provide an early line of immune surveillance and defense by ingesting and degrading viruses, releasing cytokines in response to PRR signaling, and bridging the innate and adaptive immune system as professional antigen-presenting cells. Viral infection of Mo/Mϕ themselves can provoke alterations in cytokine and chemokine expression, transcriptional response, cell motility, and differentiation to distinct or even simultaneous inflammatory and immunosuppressive polarization. Nikitina et al. provide an excellent review of these mechanisms (20).

Notably, SARS-CoV-2 is capable of directly infecting Mo/Mϕ without cytopathic effect, resulting in significant release of both pro- and anti-inflammatory cytokines including IL-6 and IL-10, with induction of CD163 in CD16-expressing monocytes, and diminished HLA-DR expression (21). Indeed, in SARS-CoV infected macaques, CD163+ macrophages are productively infected, and may act as viral reservoirs (22). Post-mortem lung tissues from human COVID-19 patients show extensive infiltration of immune cells, including abundant monocytes and macrophages. In these fatal cases, broad tropism to respiratory epithelia and immune cells is reported, with 90% of infiltrating immune cells showing positive staining for SARS-CoV-2 viral proteins. The number of infected cells is also correlated with the extent of tissue damage (23).

Alterations in IFN signaling in response to SARS-CoV-2 infection may contribute to delayed control of viral replication, coupled with dysregulated inflammatory pathology. Infection of respiratory epithelia and immune cells by SARS-CoV-2 induces expression of a subset of ISGs in an IFN-independent manner, contributing to recruitment of inflammatory Mo/Mϕ into infected tissue. In naïve *ex vivo* human lung tissue, SARS-CoV-2 infects type I and type II alveolar pneumocytes as well as alveolar macrophages, with rapid viral replication and significant expression of IL-6, CCL2/MCP-1, and CXLC10/IP-10, yet without significant induction of Type I, II, or III IFNs (24). Respiratory epithelial cells infected *in vitro* by SARS-CoV-2 show exuberant inflammatory cytokine production, coupled with weak or delayed induction of IFN-I and -III, suggesting that impaired innate defense against early viral replication and epithelial infection contributes to COVID-19 pathology. Post-mortem COVID-19 lung samples also display strong induction of a subset of ISGs, particularly monocyte associated chemokines such as CCL2/MCP-1 and CCL8/MCP-2, yet without detectable expression of IFN-I or IFN-III (25).

Human monocytes and respiratory epithelial cells, but not lymphocytes, express ACE2, which is used as a viral entry receptor by both SARS-CoV-2 and SARS-CoV. In human patients with SARS-CoV infection, increased CXCL10/IP-10 levels in immune cells and lung epithelia are induced in an IFN-independent manner, and correlate with recruitment of CD68+ monocytes into interstitial lung tissue, accompanied by progressive lymphopenia and elevated LDH, consistent with rapid recruitment and apoptosis of T-lymphocytes (26).

Similarly, infection of monocyte-derived macrophages by SARS-CoV *in vitro* induces expression of CCL2/MCP-1 and CXCL10/IP-10 in an IFN-independent manner (27). Delayed IFN-I signaling in SARS-CoV-infected mice promotes inflammatory Mo/Mϕ accumulation and impaired virus-specific T-cell responses. Exogenous IFN-I delivery prior to peak virus titer ameliorates severity, yet later IFN-I delivery exacerbates Mo/Mϕ-associated inflammation. Depletion of inflammatory Mo/Mϕ by inhibiting CCR2 (the receptor for CCL2) confers protection against lethal disease (28).

Interaction between viral glycoproteins and host lectin receptors may contribute to Mo/Mϕ infection. The SARS-CoV-2 virus is heavily glycosylated, and the S protein is recognized by several CLRs including mannose receptor CD206/MR, CD209/DC-SIGN, CD209L/L-SIGN, and CD301/CLEC10A, which are highly expressed in Mo/Mϕ. Significant co-expression of CLRs including CD206/MR, CD209/DC-SIGN, and CD301/CLEC10A, along with inflammatory cytokine and chemokine production, is observed in activated macrophages and DCs from patients with COVID-19 (29).

In addition to mediating viral recognition and downstream signaling pathways, membrane-bound receptors such as CLRs can enhance viral adhesion to target cells and may also serve as viral receptors. For example, CD209L/L-SIGN binds to SARS-CoV spike, and may serve as an alternate receptor independent of ACE2, while viral binding to cells bearing CD209/DC-SIGN allows dissemination of SARS-CoV to cells that are permissive for viral entry (30). Viral attachment to host cells may also be facilitated by binding interactions between viral envelope proteins and sialic-acid binding lectins expressed on host cells (e.g., CD169, FCN1), potentially activating endocytic and immune response pathways (31).

The “cytokine storm” associated with MAS/secondary HLH generally features sustained fever, hyperferritinemia, coagulopathy, and elevated release of inflammatory cytokines such as IL-1, IL-6, and IL-18. Macrophage activation syndrome can emerge as a severe complication in a variety of inflammatory conditions, including systemic lupus, Kawasaki Disease, and systemic juvenile idiopathic arthritis. Elevated expression of CD163 is also observed in monocytes and macrophages, which can be upregulated by IL-10, suggesting that this expression may have a compensatory role (32). These inflammatory features are consistent with those observed in COVID-19. In COVID-19 patients experiencing respiratory failure, immune responses are reported to be universally classified by either MAS (based on ferritin > 4,420 ng/ml) or immune dysregulation similar to septic immunoparalysis (based on HLA-DR on CD14 monocytes <5,000), representing about 25 and 75% of patients, respectively. In the latter group, overproduction of cytokines is combined with reduced lymphocyte count, and a decrease in HLA-DR on CD14 monocytes that is inversely correlated with IL-6 (33).

The immune response of Mo/Mϕ in severe COVID-19 shares notable characteristics with other inflammatory conditions. Zhang et al. examined single-cell RNA-seq profiles of monocytes and macrophages obtained from analysis of COVID-19 bronchoalveolar lavage fluid (BALF) and tissues from multiple inflammatory diseases including ulcerative colitis, rheumatoid arthritis, systemic lupus erythematosus, Crohn's disease, and interstitial lung disease. COVID-19 was reported to share two CD14+CD16+ inflammatory macrophage phenotypes with these diseases: one characterized by a CXCL10+CCL2+ cytokine signature, as well as a population expressing the pathogen recognition and complement lectin pathway receptor FCN1/Ficolin-1 (34). Enrichment of FCN1-expressing macrophages is observed in BALF of COVID-19 patients with severe/critical disease relative to patients with moderate infection and controls (11). FCN1 shows elevated expression in both Kawasaki Disease and rheumatoid arthritis, binds both IgG and sialic acid, and correlates with levels of autoantibodies in these conditions. Blockade of FCN1 reduces inflammation in a murine model of arthritis, suggesting that downregulation of FCN1 may be a mechanism of therapeutic intravenous immunoglobulin (35).

## Antibody Response and Immune-Enhanced Pathology

Disease severity in COVID-19 is positively correlated with antibody response. Emphatically, this relationship may reflect extensive host–virus interactions in patients with severe disease without implying a pathological role for antibodies. Still, severe disease progression is not reliably curtailed by high production of neutralizing antibodies (NAbs). Rather, severe disease is strongly associated with high levels of NAbs targeting the SARS-CoV-2 receptor binding domain (RBD) located on the trimeric S1 spike (S-RBD). NAbs targeting the S1 spike are reported to be dependent on RBD-specificity for neutralization capacity. Neutralizing antibodies specific to the S2 subunit are also observed in the majority of patient sera. Patients with severe disease generate high NAb titers, while asymptomatic patients may mount little or potentially no NAb response (36).

Based on serial blood sampling, rapid exacerbation of symptoms and progressive lymphopenia among patients with severe and critical disease are observed to coincide with IgG seroconversion, and cannot be explained by uncontrolled viral replication. In contrast, symptoms such as fever, cough, and general malaise are observed to improve in patients with mild and moderate disease prior to or independent of seroconversion (37).

Antibodies with the IgG isotype exhibit the strongest positive association with COVID-19 disease severity. Age and gender (male) are also significantly correlated with antibody levels (38). Asymptomatic individuals infected by SARS-CoV-2 are reported to develop significantly lower virus-specific IgG levels than symptomatic patients, and express significantly lower levels of pro- and anti-inflammatory cytokines (39). In contrast, patients with severe COVID-19 disease produce significantly higher IgG virus-specific NAb titers, and may also seroconvert earlier than patients with mild symptoms (40). In one study, IgG levels targeting S-RBD were reported to increase early following infection only in patients with severe disease (41).

The relationship between neutralizing IgG and severity in COVID-19 mirrors that observed in SARS. In a large-scale prospective study of SARS patients, progression to critical disease generally followed a three-phase pattern. Rapid viral replication during the first week was accompanied by systemic symptoms that gradually receded. During the second week, recurrence of symptoms and severe clinical worsening occurred simultaneously with IgG seroconversion, followed by a third phase of disease progression to acute respiratory distress syndrome (ARDS) and lymphopenia in a subset of critical patients, despite a declining viral load (42). Virus-specific IgG levels in SARS-CoV infected patients were positively correlated with disease severity, including the need for ICU admission and supplemental oxygen. Moreover, early seroconversion (day 4–15 after fever onset) was observed more frequently among patients requiring admission to ICU, compared with patients who remained seronegative. Notably, the emergence of NAbs did not confer protection against disease severe progression (43). SARS patients with a short duration of illness were more likely to be seronegative, while longer duration of illness was associated with higher patient NAb levels. Patients with early seroconversion had a markedly higher fatality rate, a shorter survival time, and greater likelihood of being over 60 years of age (44). Evidently, once the host response has shifted to a hyperinflammatory state in the presence of a high viral load, neither the emergence of antibodies nor a subsequent decline in viral load appears sufficient to halt disease progression.

Although the correlation between antibody response and disease severity in COVID-19 does not establish a causative relationship, several findings suggest that antibodies to SARS-CoV-2 can participate in maladaptive immune responses. For example, anti-spike IgG from critical COVID-19 patients induces a hyperinflammatory response in monocytes cultured to resemble primary human lung macrophages. In the presence of the synthetic RNA analog poly(I:C), anti-spike IgG triggers exuberant release of IL-1β, IL-6, TNF, and IL-10 from these cells, similar to that observed in COVID-19 patients. Moreover, in the presence of human vascular endothelial cells and platelets under flow conditions, these macrophages disrupt endothelial barrier integrity and provoke microvascular thrombosis (45). Likewise, sera from COVID-19 patients show elevated levels of products indicative of NETs which can amplify inflammation and thrombosis. Patient sera also strongly trigger healthy neutrophils to undergo NETosis (46), and IgG fractions isolated from severe COVID-19 patients induce apoptosis of platelets from healthy donors via cross-linking of FcγRIIa receptors (47), possibly contributing to immune-enhanced severity in COVID-19.

## Antibody-Dependent Enhancement of Mo/Mϕ Infection

As monocytes are susceptible to receptor-mediated infection via surface expression of ACE2 (26), the potential mechanisms of monocyte infection and immune-enhanced disease in COVID-19 are not reliant on ADE of infection. However, several viruses, including coronaviruses such as SARS-CoV, MERS-CoV, and feline infectious peritonitis virus (FIPV), are able to exploit antibodies to increase infection and expand tropism to immune cells expressing Fc receptors that recognize and bind to antibody Fc domains. For example, although vaccine-induced immune serum directed against the SARS-CoV spike neutralizes viral entry via ACE2 receptors in permissive cell lines, it also enables viral entry into Fcγ receptor-bearing human monocytes and B-lymphocyte derived cells. Infection by replication-competent SARS-CoV virus is accompanied by viral gene transcription and protein synthesis, which may alter immune cell function even though it does not proceed to viral replication. Blockade of FcγR abrogates antibody-mediated infection (48). Notably, genetic variations in CD14 and the Fcγ receptor CD32/FcγRIIa confer risk of severe SARS-CoV infection (49).

In a recent study of circulating monocytes isolated from SARS-CoV-2 infected patients presenting for emergency hospital care, 10% of monocytes were infected by the virus, including double-stranded RNA (dsRNA) staining indicative of viral replication, and markers of NLRP3 and AIM2 inflammasome activation. While monocytes derived from healthy donors were inefficiently infected by SARS-CoV-2, pre-incubation with anti-spike antibody or patient plasma was reported to enhance productive infection of monocytes. This effect was abrogated by Ig depletion of patient plasma (50). Moreover, positive- and negative-strand SARS-CoV-2 RNA is detected in alveolar macrophages recovered from BALF of intubated patients with severe COVID-19, and in monocyte-derived macrophage and DC subsets that do not express ACE2 (51).

Monocyte lineages are the primary target of ADE of SARS-CoV infection in the presence of anti-S IgG, and human macrophages are also infected. Antibody-dependent enhancement is dependent on intact intracellular signaling of domains of FcγR, but productive replication of SARS-CoV in infected macrophages is not observed (52). The close relationship between the neutralizing capacity of antibodies and their capacity for ADE of coronavirus entry is notable. A similar relationship between neutralization and enhancement is observed in FIPV, which preferentially infects Mo/Mϕ. Moreover, ADE of infection can be induced by exposure to the same viral serotype to which vaccine-induced antibodies are directed (53).

A central consideration in the evaluation of vaccine safety is the immune response to subsequent viral challenge. SARS-CoV spike-based vaccination of macaques, followed by challenge by live virus, was reported to produce fever 1–5 days after challenge, with 6 of 8 vaccinated macaques developing acute DAD within 1–5 weeks. Control animals demonstrated only mild or moderate inflammation in response to viral challenge. When purified anti-spike S-IgG from vaccinated macaques was administered to healthy animals and followed by viral challenge, all recipients showed acute DAD, with features including hyaline membrane formation, hemorrhage, and infiltration of inflammatory monocytes and macrophages. While SARS-CoV infection of control macaques induced macrophages expressing CD163 and mannose receptor CD206/MR, administration of anti-S-IgG triggered a loss of CD206/MR expression and wound-healing function of macrophages, accompanied by tissue damage and uncontrolled inflammation. Notably, the effects of vaccine-induced immunity can vary markedly depending on the animal model and vaccine design under study, and may depend partly on differences in CD8+ T cell participation and vaccine-induced Th1 response (54).

The molecular mechanism underlying ADE of coronavirus entry was recently described. Wan et al. (55) demonstrate that both MERS-CoV and SARS-CoV S-RBD-specific antibodies can effectively neutralize viral entry via DDP4 and ACE2, respectively, while also mediating viral entry into IgG Fc receptor-bearing cells.

Structurally, the coronavirus spike is comprised of three S1 subunits, each containing a RBD, connected to a trimeric S2 stalk that carries the membrane fusion mechanism. Infection of the host cell first requires cleavage of the viral S1/S2 site by host proteases such as TMPRSS2, followed by cleavage of the S2' site, which liberates viral membrane fusion mechanisms. Binding of the viral RBD to its receptor (DPP4 for MERS-CoV, ACE2 for SARS-CoV) stabilizes the bound S1 trimer in a “standing up” position, which is required in order to expose the S2' site to proteasomal cleavage. In antibody-mediated entry, the NAb binds to the tip of the viral spike where the RBD is located, and stabilizes the conformation of the S1 trimer to expose the otherwise inaccessible S2' site to cleavage (55). Binding of the Fc domain of the antibody-virus complex to membrane-bound Fc receptors on host immune cells thus allows a shift in the tropism of viral infection to FcγR-expressing cells such as monocytes and macrophages.

In ADE of SARS-CoV, enhanced entry is pH-independent and minimally affected by inhibition of endosomal proteases such as cathepsin-L, suggesting that entry may occur at the cell membrane, independent of the endocytic pathway (48). Host cell expression of the transmembrane serine protease TMPRSS2 can mediate release of the viral fusion protein to enable virus-host membrane fusion, and can also induce the formation of syncytia (large multi-nucleated cells) driven by further membrane fusion with neighboring cells (56). In contrast, ADE of MERS-CoV is reliant on lysosomal acidification and endosomal protease activity, suggesting that infection of host cells is achieved by endosomal escape. As is observed in ADE of dengue virus (DENV), ADE is strongest at intermediate levels of NAb, as low antibody levels blocked receptor-based entry to a greater degree than they encouraged ADE, and high antibody levels saturated Fc-receptor molecules (55).

Antibody-mediated entry of SARS-CoV-2 into human monocyte-derived cells and B-lymphocytes has recently been reported. Antibody-enhanced infection of these cells is mediated by engagement of FcγRII (CD32), with viral fusion occurring at the cell membrane without dependence on endocytosis. Antibody-dependent enhancement is most strongly induced by patient sera derived from elderly donors with severe disease, and is mediated by virus-specific IgG directed against S-RBD (57). SARS-CoV-2 is also reported to infect CD4+ T-helper cells, resulting in functional impairment and increased expression of IL-10. However, in contrast to ADE, infection of CD4+ cells remains dependent on the presence of ACE2. In this case, binding of the SARS-CoV-2 spike to CD4 stabilizes the virus at the cell membrane, and may help to compensate for low levels of ACE2 expression by increasing the opportunity for receptor binding (58).

The potential contribution of ADE in COVID-19 has been discussed in the context of DENV infection, for which ADE has been well-studied (59, 60). Primary dengue fever (DF) typically presents with mild symptoms, with infection of blood cells resulting in leukopenia and depressed platelet count. However, the influence of ADE in secondary DENV infections can promote severe dengue hemorrhagic fever (DHF), which is characterized by fever and often fatal vascular leakage, particularly when untreated (60). Antibody-dependent enhancement in dengue is affected by the relative abundance of FcγR isoforms having activating or inhibitory effects on immune cell activation. Activating signals are mediated by receptors carrying immunoreceptor tyrosine-based activation motifs (ITAMs) such as FcγRIIa, while inhibitory signals are mediated by receptors carrying inhibitory (ITIM) motifs such as FcγRIIb. Blockade of activating FcγRs ablates infection of cells by antibody–virus immune complexes (IC) (61).

Infants with passive immunity from DENV infected mothers typically present with DHF upon initial DENV infection. Strikingly, afucosylation of maternal anti-dengue IgG Fc domains is a highly specific predictor of symptomatic infection in infants, with afucosylated IgG in excess of 10% predictive of severe disease outcomes (61).

Core fucosylation of the IgG Fc domain modifies its binding to Fc receptors, with reduced fucosylation leading to enhanced interactions with the activating CD16a/FcγRIIIa receptor. In symptomatic adults infected with SARS-CoV-2, the Fc domains of anti S-RBD IgG antibodies are characterized by significantly reduced core fucosylation relative to IgG antibodies from healthy adults. Particularly significant reduction in fucosylation of anti-RBD IgG is observed in patients with severe disease, in comparison to patients with mild COVID-19 and asymptomatic children seropositive for SARS-CoV-2 antibodies (62).

Recombinant anti-S IgG derived from patients with severe COVID-19 characterized by low fucosylation promote increased induction of inflammatory cytokines by human macrophages (45). Critical COVID-19 ICU patients with acute respiratory distress are reported to show significantly lower levels of fucosylated anti-S IgG than mild or asymptomatic patients. Comparative analysis of immune response to multiple viruses also suggests that IgG afucosylation may be more common in response to antigens embedded in viral membranes than to non-enveloped viruses or soluble protein antigens (63).

## Self-Reactive Antibodies and FcγR Responses in Immune-Enhanced Pathology

Immune-enhanced pathology can include the induction of cross-reactive antibodies against human endothelial cells and molecules involved in platelet function and coagulation, possibly resulting from molecular mimicry by viral proteins having sequence similarities. Such autoantibodies are induced in DHF (64). Infection by Epstein Barr virus (EBV) can induce TLR hypersensitivity, followed by increased TLR-mediated B-cell differentiation to autoreactive antibody-secreting cells (65). In the presence of high levels of viral antigen, hypergammaglobulinemia and autoreactive antibody production can result from cooperation of infected B cells with CD4+ T-helper cells (66).

In patients infected with SARS-CoV-2, the generation of self-reactive antibodies has also been observed (67–69). The production of antiphospholipid antibodies of multiple isotypes has been reported in critically infected COVID-19 patients, in association with hypercoagulation and thrombotic events (70). Strikingly, nearly 30% of COVID-19 patients with severe disease, but fewer than 4% of non-intubated patients, are reported to produce IgM antibodies that cross-react with ACE2 and induce complement pathway activation. These autoreactive IgM antibodies emerge concurrent with clinical worsening and intubation, and appear only after anti-S IgG responses have been established. These autoantibodies may emerge in a T-independent manner from splenic marginal zone B cells, and could reflect an anti-idiotypic response to IgG antibodies directed against the SARS-CoV-2 spike (71).

Immune complexes comprised of IgG-bound antigens may further contribute to vascular leakage and cytokine storm via CD16/FcγRIII engagement (72). Cross-linking of FcγRs by IgG ICs induces macrophage activation and a switch in metabolic programming to glycolysis, accompanied by hypoxia-inducible factor HIF-1α dependent cytokine release, mirroring outcomes observed in IC-mediated autoinflammatory disease (73). In this context, it is notable that monocytes from patients with severe COVID-19 show high expression of HIF-1α and associated target genes, compared with healthy controls. In SARS-CoV-2 infected monocytes, mitochondrial ROS production and resulting HIF-1α-mediated metabolic changes contribute to impaired T-cell proliferation and expression of PD-1, a marker of T-cell exhaustion (74). Disruption of mitochondrial function by SARS-CoV-2 is further implicated in suppressed IFN response, NLRP3 inflammasome activation, and reduced oxygen sensing in patients with COVID-19 (75). Cross-linking of the CD16b/FcγRIIIb receptor isoform, which is present exclusively on neutrophils, also triggers the release of NETs that can contribute to thrombus formation (76).

Thus, as is observed in SARS-CoV and MERS-CoV infection, the production of antibodies against SARS-CoV-2 may potentially mediate several forms of immune-enhanced pathology, not limited to ADE, despite contributing to viral clearance. At the same time, these mechanisms suggest specific points of investigation such as ADE, core fucosylation of IgG Fc domains, induction of cross-reactive antibodies, FcγR mediated responses, compliment activation, and related factors that may be useful in evaluating the safety of candidate vaccines and antibody-based therapeutics.

## Viral Pattern Recognition Receptors in Dysregulated Cytokine Signaling

Following viral infection of immune cells, viral components such as glycosylated membrane proteins activate signaling by innate pattern recognition receptors, resulting in downstream transcription that may include dysregulated production of inflammatory cytokines and chemokines. For example, repeated activation of the ssRNA sensor TLR7 promotes Mo/Mϕ differentiation into inflammatory hemophagocytes that drive MAS-like disease (77). Notably, CD14 acts as a co-receptor for signaling by the pattern recognition receptors TLR7/9. While CD14 is dispensable for viral uptake into endosomes, it is essential for triggering inflammatory cytokine production by macrophages and DCs (78).

In MERS-CoV infected monocyte-derived macrophages, upregulation of RLR and CLRs is followed by induction of proinflammatory molecules including IL-6, CXCL10/IP-10, and CCL3/MIP-1a. Depletion of the RLR signaling adaptor MAVS (mitochondrial antiviral signaling protein) or spleen tyrosine kinase Syk significantly reduces inflammatory cytokine induction. Induction of Syk by CLR, and downstream activation of NF-κB through the CBM complex (CARD9-BCL10-MALT1) is particularly implicated in the MERS-CoV inflammatory response of macrophages (79).

Signaling by FcγRs depends on downstream signaling by Syk. Activation of PI3K by Syk induces downstream signaling to cytoskeletal proteins that mediate phagocytosis of IgG IC and receptor internalization, while additional signaling pathways promote the expression of pro-inflammatory cytokines and chemokines (80). The inflammatory response of human macrophages to anti-spike IgG from patients with severe COVID-19 is ablated by the Syk inhibitor fostamatinib, with downregulation of inflammatory mediators as well as expression of genes involved in platelet activation (45).

The inflammatory response of monocytes in SARS-CoV-2 infection shares numerous features with monocyte response in other viral infections, particularly following activation of innate pattern recognition receptors by heavily glycosylated viruses. For example, Ebola virus (EBOV) infection of monocytes results in strong induction of inflammatory cytokines including IL-6, IL-8, and chemokines CCL3/MIP-1a and CCL4/MIP-1b, with downregulation of class II MHC expression. Recruitment and apoptosis of lymphocytes is accompanied by a marked increase in LDH levels (81). Monocytes, macrophages, and DCs are initial targets of EBOV infection, and inflammatory cytokine release in EBOV-infected monocytes is mediated by TLR4 activation and downstream NF-κB signaling (82).

Likewise, infection with Hantaan virus (HTNV), which causes uncontrolled inflammatory response and lethal hemorrhage, is associated with a sharp increase in CD14+CD16+ intermediate monocytes, particularly in acute disease. Expression of CD163 in these monocytes is associated with severe disease, with CD206 expression observed more frequently in patients with mild/moderate disease (83). Viral recognition of HTNV by TLR3, RIG-I, and MDA5 pattern-receptors induces high expression of CXCL10 via NF-κB and IRF7 signal transduction pathways (84). Progression to severe hemorrhagic shock in DENV infection is also marked by upregulation of CD14+CD16+ monocytes expressing CD163 (85). DENV infection in monocytes is detected by TLR2/6, with CD14 acting as a co-receptor, resulting in the induction of pro-inflammatory cytokine expression via NF-κB signaling pathway (86).

## Discussion and Implications for Antibody-Based Therapeutics

**Figure 1:**
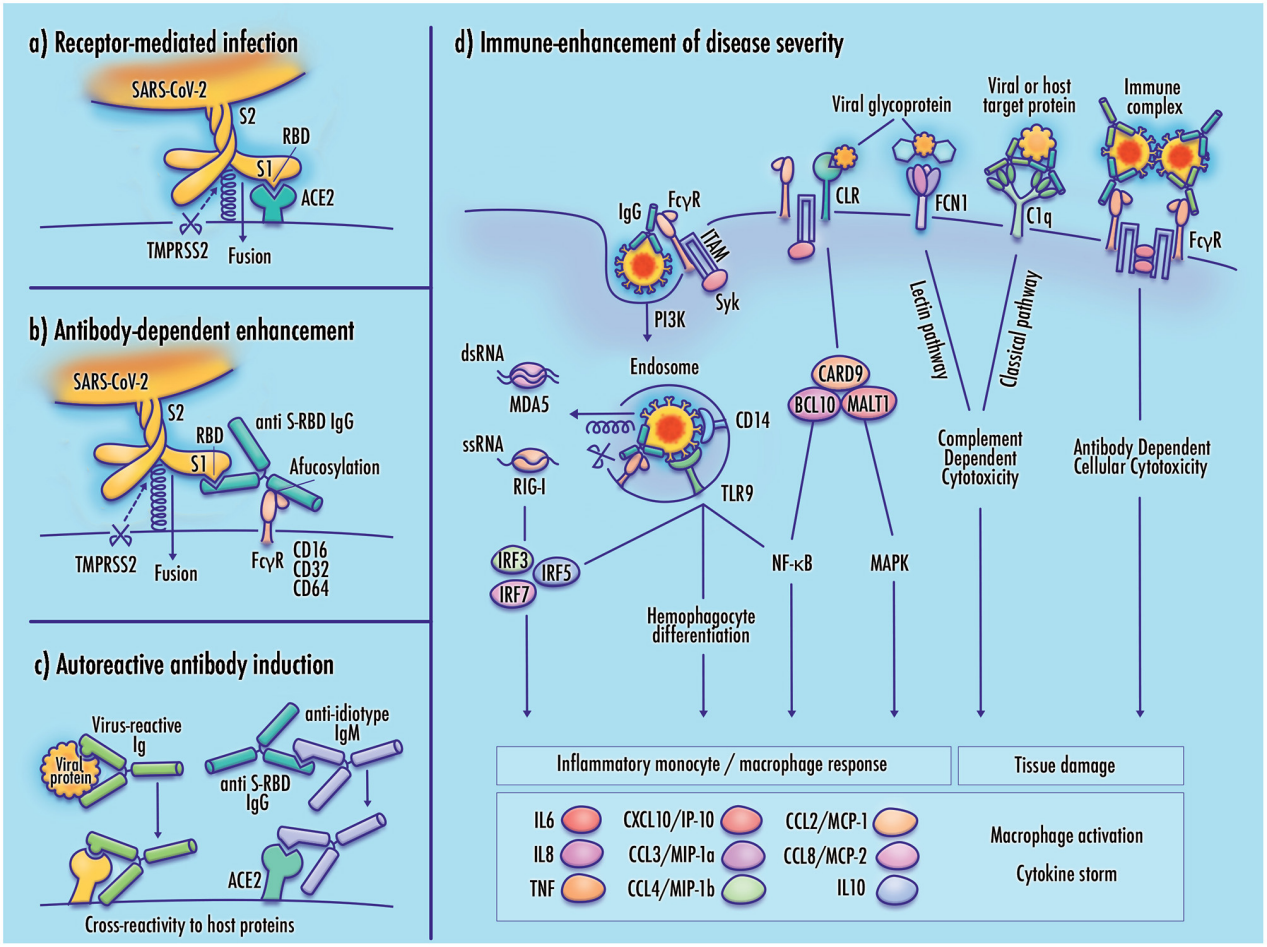
Potential mechanisms of monocyte infection and immune-enhanced severity in COVID-19. **(a)** Receptor-mediated infection of target cells by SARS-CoV-2 is achieved by binding of the viral receptor binding domain (RBD) with host membrane-bound ACE2, which allows TMPRSS2 proteasomal cleavage of the viral spike membrane to enable virus–host membrane fusion; **(b)** In antibody-dependent enhancement (ADE), binding of the virus-antibody complex to an Fc gamma receptor stabilizes the viral spike in a manner that mimics the function of the viral receptor, enabling cleavage of the viral membrane and virus–host membrane fusion; **(c)** Induction of autoantibodies may result from molecular mimicry by viral proteins having sequence similarity to host proteins, anti-idiotype antibodies with cross-reactivity to host receptors, or direct disruption of immunological tolerance, which may be induced by TLR7 hyperactivation (not shown); **(d)** Increased disease severity may result from maladaptive immune responses to the SARS-CoV-2 virus. Viral infection of monocyte/macrophages can contribute to inflammatory pathology, activating downstream cytokine signaling, and cellular differentiation pathways. Inflammatory responses may also be induced by activation of pattern-recognition receptors including RIG-I-like receptors (RLR), Toll-like receptors (TLR), and C-type lectin receptors (CLRs). Receptors expressed at the immune cell membrane mediate adhesion of viral membrane glycoproteins, potentially contributing to infectivity by stabilizing the virus at the host cell membrane. Activation of complement pathway receptors by viral glycoproteins or antibody-bound target proteins may produce tissue damage by inducing complement-dependent cytotoxicity (CDC). Cross-linking of Fc gamma receptors by immune complexes can induce antibody-dependent cellular cytotoxicity (ADCC), and the release of neutrophil extracellular traps (not shown). Elevated cytokine and chemokine expression promotes cell recruitment, increased vascular permeability, and inflammatory damage to infected tissue.

The outcomes of SARS-CoV-2 infection can range from asymptomatic presentation to critical respiratory failure, tissue damage, organ failure, and fatality. Clinical reports suggest that these outcomes do not lie along a smooth continuum, but are often marked by abrupt severe clinical worsening. It is possible that this shift toward poor clinical outcomes corresponds to a change in viral tropism from infection of cells comprising respiratory linings and alveolar epithelia to direct viral infection of immune cells such as monocytes and alveolar macrophages. Although antibody response, inflammation, complement-dependent cytotoxicity, PRR signaling, and Fc-receptor effector functions can contribute to the clearance of viral pathogens, it is possible that these responses may be dysregulated in SARS-CoV-2 infected patients in a manner that contributes to disease severity. Figure 1 illustrates several of these proposed mechanisms.

The potential roles of Mo/Mϕ infection and immune-enhanced pathology in COVID-19 are consistent with a broad range of evidence, but their prominence remains tentative pending further validation. In the interim, given the global health imperative for the development of safe and effective vaccines and therapeutics, the mechanisms discussed in this article suggest specific avenues of investigation that may be beneficial in the evaluation of candidate interventions.

For example, severe lung injury in SARS-CoV infection is not detected in macaques until 7 days following viral challenge (54), suggesting that evaluation of SARS-CoV-2 vaccine-induced ADE based on similar or shorter periods may be inappropriate. Screening for potentially cross-reactive antibodies may be informative, particularly where antigen selection includes S-RBD epitopes that overlap ACE2 binding sites. It may be useful to examine fucosylation of vaccine-induced IgG, particularly at intermediate titers in elderly or predisposed individuals, as antibody glycosylation and immune-enhanced effector functions may not be solely a property of a given vaccine or therapeutic, but also a property of the individual host response.

Currently, the majority of candidate vaccines against SARS-CoV-2 target the viral S protein, including S-RBD (87). Accordingly, the impact of spike- and S-RBD-directed antibodies on viral infection and inflammatory response of FcγR-bearing immune cells may be a particularly important focus in the evaluation of vaccine safety and efficacy. However, the potential for immune-enhanced pathology is not restricted to S-RBD epitopes or ADE of infection. For example, immunization of mice with the SARS-CoV full-length nucleocapsid protein can provoke pulmonary inflammation and immune cell infiltration upon viral challenge, despite reduction of viral titer to negligible levels (88).

As antibodies directed against the SARS-CoV and MERS-CoV S-RBD can functionally mimic the viral receptor and enable transition to a post-fusion conformation (89), inclusion of non-RBD epitopes may be advantageous. Antigen designs including modifications to stabilize the coronavirus spike in a pre-fusion conformation limit reliance on the S-RBD, concealing its immunodominant but poorly-conserved receptor binding motif (RBM), and are reported to increase both the breadth and potency of NAb responses (90, 91). Notably, the pre-fusion conformation does not prevent immune access to conserved epitopes at the periphery of the RBD (92) and at the S2 hinge (93), which mediate potent neutralization of both SARS-CoV and SARS-CoV-2.

Even after the development of initial vaccines against SARS-CoV-2, continued research and development efforts will remain important. While the coronavirus S-RBD is highly immunogenic, it is capable of readily generating antibody escape mutations in response to immune pressure (94). Adaptive S-RBD mutations have also been described after serially passaging the SARS-CoV-2 virus through the respiratory tract of aged mice. One recovered strain, carrying an N501Y substitution in the RBM, displayed enhanced ACE2 binding and replication in the respiratory tract of aged BALB/c mice, with subsequent tissue infiltration by inflammatory cells, including activated CD163+ macrophages (95). Such mutations in the RBD have recently emerged in novel strains first identified in the United Kingdom and South Africa, conferring greater resistance to neutralization by antibodies to ancestral strains (96). Prefusion spike vaccine designs and targeting of conserved epitopes may reduce this risk. Antibodies specific to a variable loop region of the betacoronavirus S2 spike subunit induce similar escape mutations in MERS-CoV, suggesting that such epitopes might best be excluded from SARS-CoV-2 vaccine designs (90).

The durability of protection conferred by vaccination against SARS-CoV-2 will likely become an active focus of research. While B-cell responses and NAbs to SARS-CoV decline significantly 1–2 years after infection, induction of memory CD4+ T-cells is suggested to confer more durable protection (97). As optimal protection against SARS-CoV-2 may rely on both antibody and T cell-mediated immunity, inclusion of highly conserved epitopes of structural or functional proteins may help to elicit a broad and durable immune response (98). Notably, much of the antibody response induced by the ChAdOx1 vaccine, encoding the full-length spike antigen without pre-fusion stabilization, appears directed toward the RBD. Three mutations in the RBD harbored by the B.1.351 variant (K417N, E484K, and N501Y) result in a loss of antibody neutralization, with reduced protection against mild-to-moderate disease. However, ChAdOx1 vaccination also elicits expansion of CD4+ and CD8+ T cells specific to a large number of spike-specific antigens, most of which are unaffected by B.1.351 mutations (99). Considerations related to antibody escape mutations and T-cell mediated-immunity may become increasingly important over time, as the combination of intermediate NAb levels with an altered future serotype may create a potentially relevant context for ADE. Although this risk remains speculative at present, careful deliberation may nonetheless be appropriate for vaccine designs that rely heavily on presentation of the viral RBD, such as chimeric S-RBD constructs.

In the event that immune enhancement is observed in a given subgroup in response to viral exposure, such as individuals predisposed to autoimmune or inflammatory response, identification of therapeutic alternatives, and mitigation strategies for at-risk individuals may be beneficial. Such options may include the use of monoclonal antibodies, potentially with Fc-domain mutations to disrupt FcγR crosslinking (100); blockade of FcγR or PRR signaling via Syk or NF-κB pathways (45, 79); downregulation of NLRP3 inflammasome activation (101); blockade of the terminal complement pathway (102); and saturation of FcγRs (55, 103) or downregulation of the complement pathway receptor FCN1 (35) via therapeutic IVIG, possibly excluding afucosylated or activating fractions. Meanwhile, ongoing consideration and testing of pathway-informed, well-tolerated therapeutic candidates may be beneficial in active cases, including repurposed therapeutics targeting viral replication (e.g., remdesivir, ivermectin), leukocyte-mediated tissue damage (e.g., doxycycline, IFN-λ), and dysregulated inflammatory response (e.g., barcitinib, ruxolitinib, tocilizumab, dexamethasone) (104) in COVID-19.

## Data Availability Statement

The original contributions presented in the study are included in the article/supplementary material. Further inquiries can be directed to the corresponding author/s.

## Author Contributions

The author confirms being the sole contributor of this work and has approved it for publication.

## Conflict of Interest

The author declares that the research was conducted in the absence of any commercial or financial relationships that could be construed as a potential conflict of interest.
